# Retraction: Molecular mechanism of inhibitory effects of melatonin on prostate cancer cell proliferation, migration and invasion

**DOI:** 10.1371/journal.pone.0342720

**Published:** 2026-02-12

**Authors:** The PLOS One Editors

After this article [1] was published, concerns were raised about Figs 2-4 and 6, and the underlying data provided in the Supporting Information. Specifically,

- The underlying image data published in the Supporting Information of this article are incomplete and some images do not appear to match the corresponding panels presented in Figs 2B, 6A, 6C, or 6E. Upon editorial follow-up, first author AN indicated that these differences in appearance arose owing to loss of quality in the stored raw data files.
- In the p50 panel of Fig 6E there appears to be a vertical discontinuity between lanes 4 and 5. Upon editorial follow-up, first author AN stated this arose owing to a natural boundary between adjacent areas of the same membrane, citing uneven exposure and scanner shading during image acquisition. They stated that all lanes were run on the same gel and belong to the same continuous membrane, and that no lanes were added, removed, or rearranged. In the absence of the raw data, this issue could not be fully resolved.
- The following microscopy images in this article [1] appear to fully or partially overlap:
  - The Fig 3A 0.5 mM and 0.8 mM 0 h panels
  - The Fig 3A 0 mM, 0.5 mM, and 0.8 mM 24 h panels
  - The Fig 3B 0.5 mM 24 h panel and the Fig 4B Scramble + melatonin 24 h panel
  - The Fig 3B 0.5 mM 48 h panel and the Fig 4B Scramble + melatonin 48 h panel
  - The Fig 3E 0.5 mM Du145 panel and the Fig 4E Scramble (-) Du145 and SiIL2RB (-) Du145 panels
  - The Fig 3E 0.8 mM Du145 panel, and the Fig 4E SiIL2RB (+) Du145 and SiIL2RB (+) PC3 panels
  - The Fig 3E 0mM PC3 panel and the Fig 4E Scramble(-) PC3 panel
- Editorial assessment of the individual-level data provided in the S1 File of [1] raised concerns about the normalization procedure of these data. In addition, PLOS identified discrepancies between the data provided in the Supporting Information files and their corresponding panels in the published figures.

First author AN acknowledged the panel overlaps observed in Figs 3 and 4 and provided replacement data and underlying microscopy image files, along with a quantitative analysis file for the western blot quantification data. Editorial assessment of the underlying and replacement data identified additional concerns about the reliability of the image data.

In light of the above concerns, which question the reliability and integrity of reported results and conclusions, the *PLOS One* Editors retract this article.

AN and MAK did not agree with the retraction. XZ and HCC either did not respond directly or could not be reached.
